# A Study on the Clustering of Daily Fruit and Vegetable Consumption by Educational Level in European Region Countries

**DOI:** 10.1002/fsn3.70510

**Published:** 2025-06-27

**Authors:** Mustafa Enes Işıkgöz, Mahir Arslan

**Affiliations:** ^1^ Rectorate Department Sakarya University Serdivan Sakarya Province Turkey; ^2^ Department of Nutrition and Dietetics, Faculty of Health Sciences Sivas Cumhuriyet University Sivas Turkey

**Keywords:** cluster analysis, daily consumption, dietary habits, educational level, European region countries, fruits and vegetables

## Abstract

The study analyzed 2022 Eurostat data on the daily consumption of fruit and vegetables (FV) in 31 European countries, categorized by educational level (ISCED 2011: 0–2, 3–4, 5–8). Consumption was categorized into “0 portions,” “1–4 portions,” and “5+ portions” for adults aged 15+. K‐means clustering (an unsupervised machine learning algorithm used to categorize the data into clusters), discriminant analysis (IBM SPSS Statistics 27, JASP 16.4), and one‐way ANOVA were applied to classify four different country groups. The robustness of these clusters was assessed using Box's *M* test (a test for equality of covariance matrices), with results indicating no significant difference (*p* > 0.05). K‐means clustering grouped the countries based on similar FV consumption patterns, while discriminant analysis and ANOVA (*F*‐test) validated the clusters and found significant differences between them. The results showed that “0 portions” and “1–4 portions” were influential in determining the clusters across all education levels (*p* < 0.001), while “5+ portions” showed no significant relationship. Further analysis by education level revealed that “0 portions” had the greatest association with lower levels of education (level 0–2; *F*(3,27) = 13.168, *p* < 0.001), while “1–4 portions” was more influential with higher levels of education (level 5–8; *F*(3,27) = 19.380, *p* < 0.001). Clustering focused on total FV consumption, not on specific portions. Countries with similar socioeconomic and educational characteristics clustered together, indicating common dietary habits. The study shows the role of education in determining FV consumption and highlights the regional differences in diets across Europe.

## Graphical abstract

This study used 2022 Eurostat data to cluster 31 European countries by daily fruit and vegetable consumption and educational level. K‐means clustering revealed that countries with similar education and socioeconomic status have similar dietary habits. The results highlight the strong association between lower education and low fruit and vegetable intake, emphasizing the role of education in healthy eating patterns across Europe.

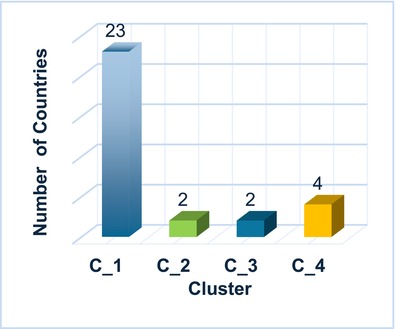

## 1 Introduction

Nutrition, recognized as one of the basic physiological needs for human survival, plays a central role in every phase of the life cycle. It is a complex process in which the human body skillfully utilizes the nutrients and bioactive compounds in food to sustain life, promote robust growth and development, improve physical and mental health, enhance quality of life, and ensure maximum productivity (Mann and Truswell 2007). Recent studies have also emphasized the importance of nutrients and bioactive compounds in supporting metabolic health and preventing chronic diseases (Abduallah et al. 2023; Choudhary and Tahir 2023). In addition, lactoferrin, a bioactive compound from milk, has shown promising health benefits and potential applications in functional foods (Hussain et al. 2024). Nutrients fulfill three essential functions: They are the foundation for growth and development, serve as the driving force for providing energy, and play a role in regulating intricate metabolic processes (Costill 2010). This assertion is supported by compelling evidence: The well‐being of individuals is critical to a healthy life and inextricably linked to the economic progress of society. An adequate and balanced diet, which is synonymous with optimal nutrition, is a cornerstone of optimal health and holistic well‐being (Pekcan 2022). The concept of “health” is interpreted differently by different institutions and individuals. The World Health Organization (WHO), for example, defines health as a state of complete physical, mental, and social well‐being and not merely the absence of disease or infirmity (Misselbrook 2014). This definition emphasizes that true health encompasses overall well‐being in multiple dimensions and not just the absence of disease.

Nutrition is an important global issue. With the United Nations' ambitious 2030 Sustainable Development Goals (SDGs), the world is united in a common mission to eradicate poverty, protect our precious planet, and ensure prosperity for all, while decisively eradicating the scourge of hunger and malnutrition. Among the pillars of these aspirations, improved nutrition is proving to be a crucial linchpin for success. For example, SDGs 2 and 3 clearly emphasize the importance of FVs not only for combating nutritional deficiencies, but also for strengthening our immune system and preventing non‐communicable diseases, as Tsalis et al. (2020) point out. Nutrients, the building blocks of life, are categorized according to the amount required for human growth and physical well‐being. They include macronutrients: proteins, carbohydrates and fats, and micronutrients: vitamins and minerals. While robust fats and carbohydrates provide energy, proteins, the structural and functional units found in every cell, drive cell growth and development. At the same time, vitamins and minerals support your body's internal processes by maintaining important metabolic functions, as Zohoori and Duckworth (2020) note. Conscientious intake of macro and micronutrients in balanced amounts and at the right time proves to be a key concern in protecting our health.

This study examines the daily consumption habits of FVs, which have become the focus of national and international discussions on healthier and more sustainable eating habits. Consistent and sufficient consumption of these nutrient‐rich FVs across all life stages is an indispensable pillar of not only a balanced, but a truly vital and healthy diet (Carrara and Schulz 2018; Kähkönen et al. 2020). Remarkably, there is a very strong, positive correlation between the consumption of these profoundly healthy food groups and the overall health of the population. Furthermore, a wealth of research clearly supports and underpins the notion that consuming sufficient amounts of these nutrient‐rich FVs is absolutely essential for achieving and maintaining optimal health and holistic wellbeing (McKenzie et al. 2020; Peltzer and Pengpid 2017; Sapranaviciute‐Zabazlajeva et al. 2017). Not only are these natural powerhouses a rich source of essential, life‐sustaining vitamins and minerals for human health, but they also provide a wide range of potent, health‐promoting phytochemicals that significantly improve overall well‐being.

The WHO and the Food and Agriculture Organization of the United Nations (FAO) have clearly identified inadequate consumption of FV as a major risk factor contributing significantly to global mortality. They strongly recommend that adults should consume at least 400 g of FV daily to promote overall health and combat the threat of non‐communicable diseases. These recommendations are widely recognized as important and play an indispensable role in combating lifestyle‐related chronic diseases and promoting robust overall health (FAO and CIRAD 2021; The European Food Information Council 2020; WHO 2002). Furthermore, the original text appears to be incomplete and lacks meaningful clarity. With visionary foresight, the United Nations General Assembly has declared 2021 the International Year of Fruit and Vegetables (IYFV). This initiative has been strategically designed to shine a spotlight on this important sector and provide an in‐depth and comprehensive perspective on an approach to production and consumption that not only makes for healthier people, but also promotes a healthier environment (FAO 2020).

Compared to the lifestyles and eating habits of past centuries, the modern world is at a crossroads characterized by profound changes (Di Daniele 2019). This pressing problem represents a major global challenge. Malnutrition and undernourishment are among the greatest global challenges facing our society (Global Nutrition Report 2022). These key factors determine the course of food choices and eating habits. Socioeconomic status and income in particular have a significant influence on food choices and eating habits. A large proportion of the world's population is unable to consume the recommended amount of FVs due to economic constraints. This widespread malnutrition is a serious obstacle to global health (FAO and CIRAD 2021). There is growing evidence that as socioeconomic status increases, the consumption of animal foods, fats and sugars increases, while the consumption of cereals, vegetables, and fruits decreases (Cagna‐Castillo et al. 2023; Jiang et al. 2022).

Beyond the undeniable influences of socioeconomic status and income, education emerges as a paramount and transformative catalyst that profoundly shapes an individual's ability to develop robust and balanced eating habits from an early age (Colley et al. 2018; Jarpe‐Ratner et al. 2016; Matwiejczyk et al. 2018). In this context, it is important that nations allocate resources to health and nutrition education programs for children. These efforts must go hand in hand with strategic investments in the health sector, with the clear aim of combating disease and improving nutritional status (Tolossa et al. 2020). Such programs play a critical role in promoting healthy eating habits among children, both within and beyond the confines of the school environment. This impact is particularly compelling as it relates to the remarkable decline in childhood obesity evidenced by transformative studies (Jung et al. 2019; Leis et al. 2019).

Nutrition programs, carefully embedded in the educational system play an important role in forming healthy eating habits in children, both at school and in their everyday lives outside the classroom. This link is notable in that it can reduce the risk of childhood obesity—a benefit that is supported by research (Jung et al. 2019; Leis et al. 2019). Experts advocate providing basic nutrition education to adolescents through well‐structured school programs (Hayes et al. 2018). Acquiring the habit of eating fresh FVs during these critical periods of childhood and adolescence proves to be an important protective measure, not only against the risk of childhood obesity, but also as a formidable shield against a variety of diseases that can occur later in life (Banna et al. 2020; Keya et al. 2019; WHO 2012). The evidence is clear: there is a strong link between healthy consumption of fresh FVs in the early years of life and a significantly lower risk of obesity and related health problems in adulthood.

This study examines the consumption patterns of FVs in 31 European countries, stratified by educational level, using cluster analysis methods. The analysis focuses on nationally representative data from 2022, expressed as a percentage of the population by education level. Although previous research has shown a link between education and dietary habits, there are still significant gaps in understanding regional differences across Europe, particularly in the context of nutrition policies aligned with the SDGs. Our study fills this gap by conducting a comparative analysis of Eastern and Western European countries and assessing the role of education as a social determinant of dietary behavior—an aspect that has not been sufficiently explored in the current literature.

## 2 Method

### 2.1 Study Group

This study covers 31 European countries and provides a comparative analysis of their key indicators. The countries analyzed are: Austria, Belgium, Bulgaria, Croatia, the Republic of Cyprus, the Czech Republic, Denmark, Estonia, Finland, France, Germany, Greece, Hungary, Iceland, Ireland, Italy, Latvia, Lithuania, Luxembourg, Malta, the Netherlands, Norway, Poland, Portugal, Romania, Serbia, Slovakia, Slovenia, Spain, Sweden, and Turkey.

### 2.2 Data Set

The data set used for this study was taken directly from the Eurostat database (Eurostat 2022). Table 1 provides a detailed summary of the dataset, including all relevant variables and measurements. The data collected by Eurostat as part of the European Health Interview Survey (EHIS) are systematically divided into four different modules: Health status, Health determinants, Health care dynamics, and a set of background variables, all covering the socio‐demographic profiles of the population. This invaluable data plays a central role in accurately and thoroughly measuring the health status of European Union (EU) citizens, including key aspects of disability, health determinants focusing on lifestyle choices, and barriers to accessing healthcare, all in a harmonized format that ensures a high degree of comparability between different Member States (MS) (Eurostat 2022). The dataset used for this study includes indicators of daily FV consumption from the health determinants module, which covers a range of individual and environmental health determinants.

**TABLE 1:** Data set information.

Online data code	hlth_ehis_fv3e
Source of data	Eurostat
Last data update	05/04/2022
Last structure update	Eurostat 04/01/2024
Overall data coverage	2014–2019
Number of values	39,708
Data navigation tree location population and social conditions > Health > Health determinants > Consumption of fruits and vegetables	

*Note:* Source of data Eurostat. https://doi.org/10.2908/hlth_ehis_fv3e.

This comprehensive dataset contains detailed indicators of daily FV consumption across 31 countries among individuals aged 15 years and older, categorized by country of residence, household characteristics, gender, age group, and educational background. The consumption indicators are categorized into three groups: 0 portions, 1–4 portions, and 5 or more portions per day. The analysis examines daily FV consumption patterns by education level.

Education levels are clearly categorized according to the International Standard Classification of Education (ISCED) 2011 standards: levels 0–2, covering education up to middle school, represent basic educational attainment; levels 3–4 represent post‐middle school and post‐secondary education (but not higher education), while levels 5–8 correspond to higher education (UNESCO 2012). Table 2 presents the proportion of each country's population consuming FVs daily, stratified by education level. This analysis shows dietary patterns and their relationship with education level.

**TABLE 2:** Daily consumption of fruits and vegetables by educational levels.

Country	0 portions (%)			1–4 portions (%)			5+ portions (%)		
	L0‐2	L3‐4	L5‐8	L0‐2	L3‐4	L5‐8	L0‐2	L3‐4	L5‐8
Austria	35.90	38.00	31.80	60.20	57.30	60.00	3.80	4.70	8.20
Belgium	20.60	19.70	12.60	69.00	67.20	67.10	10.40	13.10	20.30
Bulgaria	54.90	48.30	36.80	41.20	46.80	56.80	3.90	4.90	6.40
Croatia	30.30	29.50	23.70	60.60	60.30	66.10	9.10	10.10	10.20
Cyprus	34.90	36.50	28.40	58.90	56.80	61.20	6.20	6.70	10.40
Czechia	54.10	49.70	39.30	40.40	42.80	50.90	5.60	7.50	9.80
Denmark	49.80	44.50	28.60	32.70	37.40	41.80	17.50	18.10	29.60
Estonia	47.90	41.10	32.70	43.10	46.60	50.50	9.00	12.30	16.80
Finland	43.60	44.00	25.50	44.90	43.80	58.10	11.50	12.20	16.50
France	25.60	27.40	23.20	56.70	52.90	54.40	17.60	19.70	22.30
Germany	34.30	35.20	27.60	55.50	54.60	59.30	10.20	10.30	13.10
Greece	38.90	36.90	20.70	53.40	51.30	60.40	7.60	11.80	18.90
Hungary	38.80	36.40	34.20	55.80	55.80	54.90	5.40	7.80	10.90
Iceland	36.20	35.70	30.40	55.80	57.10	57.20	8.00	7.20	12.40
Ireland	25.00	20.60	13.30	50.60	46.70	47.60	24.40	32.70	39.10
Italy	24.70	24.40	19.60	66.00	64.60	67.20	9.30	10.90	13.20
Latvia	66.90	55.20	44.00	28.60	39.20	44.40	4.50	5.60	11.70
Lithuania	49.60	44.60	33.10	38.70	40.80	47.70	11.70	14.60	19.20
Luxembourg	52.80	54.40	41.60	35.80	34.80	41.30	11.40	10.80	17.10
Malta	38.70	39.00	30.20	52.00	49.90	53.50	9.30	11.10	16.30
Netherlands	43.10	46.00	35.10	31.60	28.20	26.90	25.30	25.80	38.00
Norway	34.30	30.90	24.40	60.20	61.50	64.20	5.60	7.60	11.40
Poland	46.80	39.10	28.10	48.50	52.80	59.50	4.70	8.00	12.40
Portugal	27.80	31.20	21.80	59.40	56.80	56.30	12.80	12.00	22.00
Romania	80.50	73.20	60.60	17.80	24.50	35.40	1.70	2.40	4.00
Serbia	50.70	45.10	35.60	43.70	47.30	51.90	5.60	7.60	12.50
Slovakia	48.70	42.20	34.20	45.10	49.30	56.00	6.10	8.50	9.80
Slovenia	30.70	31.00	32.50	64.80	65.00	61.60	4.50	4.00	5.90
Spain	25.60	23.40	19.90	64.50	66.00	67.40	9.90	10.60	12.80
Sweden	42.40	40.60	29.90	51.00	53.20	60.30	6.60	6.30	9.80
Turkey	38.80	37.40	33.70	58.20	60.10	63.60	3.00	2.60	2.70

*Note:* Table design and data presentation: Authors; Data source: https://ec.europa.eu/eurostat/databrowser/view/HLTH_EHIS_FV3E$DEFAULTVIEW/default/table.

Table 3 contains descriptive statistics on daily FV consumption in 31 countries, stratified by educational level. The most pronounced mean (*M* = 41.06, 95% CI [37.15–45.70], SD = 13.09) in the group that does not consume FVs daily (0 portions) is observed in the lower education levels (0–2). In contrast, the highest mean (*M* = 54.95, 95% CI [51.45–57.84], SD = 9.59) is observed in the higher education levels (5–8). It is noteworthy that the highest consumption is observed among those who consume 5 or more portions of FVs daily, with the mean (*M* = 14.95, 95% CI [12.11–18.31], SD = 8.51) being highest in grades 5–8. These results underline the relationship between educational level and dietary habits.

**TABLE 3:** Means of daily consumption of fruits and vegetables according to educational levels.

	Mean (95% CI)	SD	Minimum	Maximum
0 portions				
Levels 0–2	41.06 (37.15–45.70)	13.09	20.60	80.50
Levels 3–4	38.74 (35.33–42.91)	11.09	19.70	73.20
Levels 5–8	30.10 (27.18–33.42)	9.36	12.60	60.60
1–4 portions				
Levels 0–2	49.82 (45. 45–53. 49)	12.29	17.80	69.00
Levels 3–4	50.69 (46.87–53.94)	10.71	24.50	67.20
Levels 5–8	54.95 (51.45–57.84)	9.59	26.90	67.40
5+ portions				
Levels 0–2	9.10 (7. 17–11. 20)	5.66	1.70	25.30
Levels 3–4	10.56 (8.41–12.93)	6.45	2.40	32.70
Levels 5–8	14.95 (12.11–18.31)	8.51	2.70	39.10

### 2.3 Statistical Analysis

The researchers used the non‐hierarchical K‐Means clustering analysis developed by MacQueen in combination with the Euclidean distance metric to identify countries with similar daily fruit consumption and to examine consumption patterns by education level. The K‐Means method is the leading titan of non‐hierarchical clustering analysis and is among the most elegant and simple but widely used, unsupervised machine learning algorithms (Cohn and Holm 2021; Giordani et al. 2020). Within the complicated framework of the algorithm, classification is performed by skillfully positioning data points near the cluster centers with which they have the closest or most similar association (Sinaga and Yang 2020). These algorithms make the grandiose assumption that clusters are inherently hyperellipsoidal and have analogous dimensions, a notion that is unequivocally reiterated (Bramer 2020). In the following section, we will present the results of this robust analysis.

To reduce the distortion in the analysis caused by outliers, the data was standardized with Z‐scores. In K‐means clustering, it is important to select the right variables and the optimal number of clusters to achieve meaningful and accurate results. The equation *k* = (*n*/2) (a practical approach proposed in the literature: Han et al. 2022; Kodinariya and Makwana 2013) was used to determine the optimal number of groups. Where n is the number of observations and k is the number of clusters. The results of this equation were further validated by comparing them with the Elbow method using the sum of squares (WSS), the Akaike Information Criterion (AIC), the Bayesian Information Criterion (BIC), and the Calinski‐Harabasz Index (CIH) (Akaike 1987; Caliński and Harabasz 1974; Schwarz 1978).

In order to determine whether there are statistical differences between the clusters selected on the basis of the optimal number of clusters, the homogeneity of variance was first tested using the Levene test. Subsequently, the mean values of the variables with consistent distributions at the cluster level were analyzed using analysis of variance (ANOVA) and *F*‐tests. The Welch test was used if the variables deviated from homogeneous distribution paths. In cases where the variables deviated from homogeneous distribution paths, the robust Welch test boldly stepped into the limelight. All statistical analyses were performed using SPSS (version 27.0; IBM Corp 2020) and JASP (version 16.4; JASP Team 2024).

## 3 Results

**FIGURE 1:**
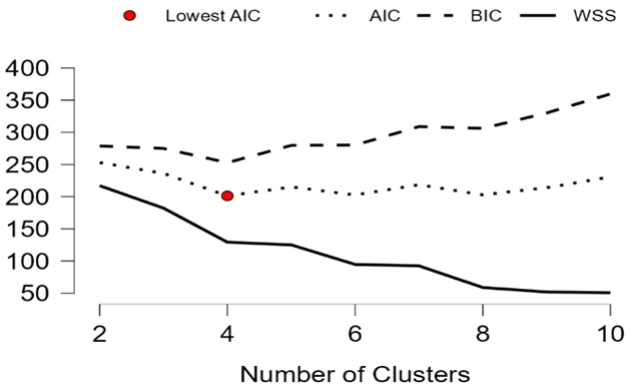
Elbow method.

**FIGURE 2:**
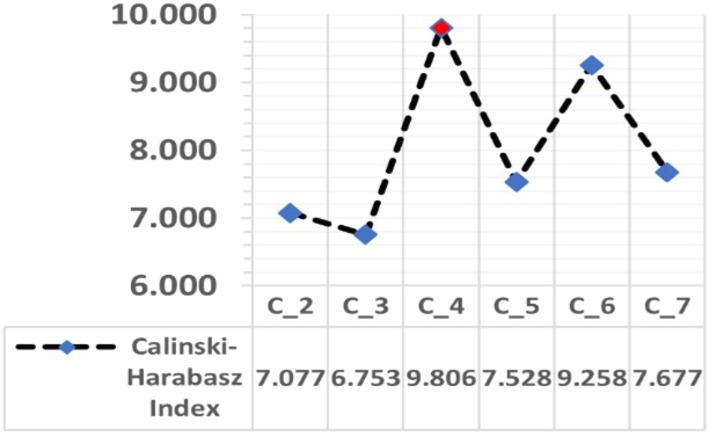
Calinski Harabasz index.

The study found that the optimal number of clusters for the analysis is four, a conclusion supported by the equation *k* = (31/2) 3.937. Furthermore, the WSS curve showed a kink point, and the lowest AIC and BIC values also favored the four‐cluster solution (Figure 1). In stark contrast, the authoritative CHI, a key tool for determining the optimal number of clusters within the K‐Means clustering algorithm, showed that this critical index value continues to decrease after the fourth cluster (CHI = 9.806). This strongly suggests that the best cluster configuration should indeed be four (Figure 2).

**FIGURE 3:**
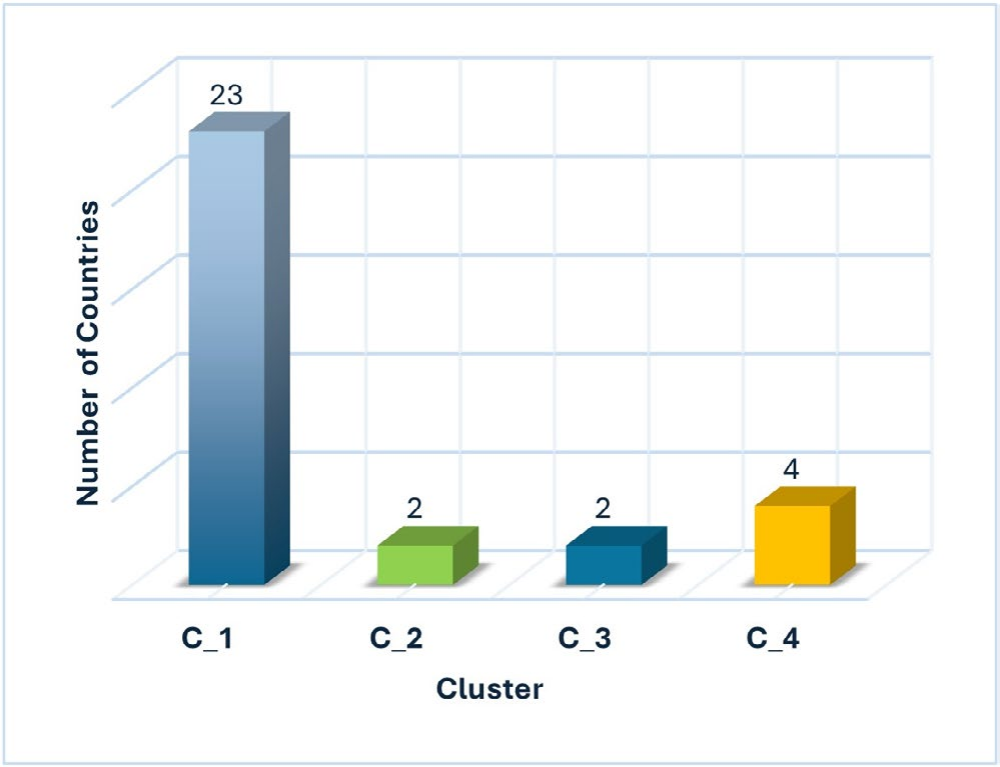
Countries by cluster.

An examination of the distribution of countries within the clusters (Table 4) shows that an impressive 23 countries (Austria, Belgium, Bulgaria, Croatia, Cyprus, Czech Republic, Estonia, Finland, Germany, Greece, Hungary, Iceland, Italy, Malta, Norway, Poland, Portugal, Serbia, Slovakia, Slovenia, Spain, Sweden, and Turkey) belong to Cluster 1. Two countries (France and Ireland) claim their residence in cluster 2, two further countries (Latvia and Romania) find their place in cluster 3 and four countries (Denmark, Lithuania, Luxembourg and the Netherlands) are clearly assigned to cluster 4. The distribution of the countries across the clusters is shown in Figure 3.

**TABLE 4:** Countries clustered according to the K‐means method.

Clusters	Countries included in clusters	The number of countries in each cluster
C_1	Austria, Belgium, Bulgaria, Croatia, Cyprus, Czechia, Estonia, Finland, Germany, Greece, Hungary, Iceland, Italy, Malta, Norway, Poland, Portugal, Serbia, Slovakia, Slovenia, Spain, Sweden, Turkey	23
C_2	France, Ireland	2
C_3	Latvia, Romania	2
C_4	Denmark, Lithuania, Luxembourg, Netherlands	4

When looking at the final cluster centers in Table 5, it becomes clear that the countries within C_3 (Latvia, Romania) and C_4 (Denmark, Lithuania, Luxembourg, Netherlands) have the lowest daily consumption patterns of FV. In stark contrast, the areas grouped in C_2 (France, Ireland) have the highest consumption rates. The bastions with the highest daily FV intake within the 1–4 portions, regardless of education level, are located in cluster C_1. The countries with the lowest consumption rates, on the other hand, are in cluster C_3. The constellation of countries in C_1 (Austria, Belgium, Bulgaria, Croatia, Cyprus, the Czech Republic, Estonia, Finland, Germany, Greece, Hungary, Iceland, Italy, Malta, Norway, Poland, Portugal, Serbia, Slovakia, Slovenia, Spain, Sweden, and Turkey), which have the highest daily consumption in the 1–4 portions category across all levels of education, while C_3 (Latvia, Romania) has the lowest consumption. Clusters C_2 (France, Ireland) and C_4 (Denmark, Lithuania, Luxembourg, Netherlands), which consume five or more portions per day, represent the zenith of national consumption across all education levels. In stark contrast, C_3 (Latvia, Romania) remains the cradle of the leanest consumption figures.

**TABLE 5:** Final cluster centers.

	C_1	C_2	C_3	C_4
0 portions				
Levels 0–2	−0.21535	−1.20452	2.49435	0.59333
Levels 3–4	−0.21927	−1.33028	2.29569	0.77811
Levels 5–8	−0.17973	−1.26579	2.37136	0.48068
1–4 portions				
Levels 0–2	0.37547	0.31091	−2.16677	−1.23103
Levels 3–4	0.41005	−0.08312	−1.75889	−1.43681
Levels 5–8	0.45384	−0.41202	−1.56938	−1.61890
5+ portions				
Levels 0–2	−0.31710	2.10219	−1.06078	1.30261
Levels 3–4	−0.30443	2.42295	−1.01727	1.04764
Levels 5–8	−0.31352	1.85072	−0.83567	1.29522

When examining the distances measured between the cluster centers (Table 6), it was found that the closest proximity exists between clusters C_1 and C_4, with a distance of 4.046. This proximity indicates a remarkable similarity between the countries in these clusters. In contrast, clusters C_2 and C_3 are separated by a distance of 8.911, indicating a clear divergence and dissimilarity between the countries included in them.

**TABLE 6:** Distances between the final cluster centers.

Cluster	C_1	C_2	C_3	C_4
C_1	—	4.729	6.064	4.406
C_2	—	—	8.911	4.386
C_3	—	—	—	4.986

The results of the discriminant analysis showed a classification rate of 100%, which means that all cases were correctly classified (Box's *M* = 18.846, *F*(0.05, 6,156,073) = 1.867, *p* > 0.05). This correct classification rate is shown in Table 7 for reference.

**TABLE 7:** Result of the discriminant analysis.

Classification results^a^	Cluster	Predicted group membership				Total
		1	2	3	4	
Original						
Count	C_1	23	0	0	0	23
	C_2	0	2	0	0	2
	C_3	0	0	2	0	2
	C_4	0	0	0	4	4
%	C_1	100.0	0.0	0.0	0.0	100.0
	C_2	0.0	100.0	0.0	0.0	100.0
	C_3	0.0	0.0	100.0	0.0	100.0
	C_4	0.0	0.0	0.0	100.0	100.0

^a^ 100.0% of the original cases grouped correctly.

A cluster analysis was performed, followed by an ANOVA to determine statistically significant differences between the clusters. The results of the Levene's test, shown in Table 8, indicate that the statistical value for daily consumption of just under 0 portions and a modest 1–4 portions of FVs across all education level segments is below the critical benchmark of *F* (3, 27) = 2.298, but above the significance threshold of *p* > 0.05 (95% CI). For individuals consuming five or more portions of FVs per day, the Levene statistic was *F* (3, 27) = 2.298, with a significance level of *p* < 0.05.

**TABLE 8:** The homogeneity test of variances.

	Levene statistic	df1	df2	Sig.
0 portions				
Levels 0–2	1.934	3	27	0.148
Levels 3–4	0.847	3	27	0.480
Levels 5–8	0.732	3	27	0.542
1–4 portions				
Levels 0–2	1.901	3	27	0.153
Levels 3–4	0.823	3	27	0.493
Levels 5–8	0.601	3	27	0.620
5+ portions				
Levels 0–2	3.730	3	27	0.023
Levels 3–4	4.688	3	27	0.009
Levels 5–8	3.836	3	27	0.021

The ANOVA results presented in Table 9, show that habitual consumption of 0 and 1–4 portions of FV has a statistically significant association with group differences across all education levels (*p* = 0.001). In contrast, habitual consumption of 5 or more portions of FV showed no significant correlation with the group analysis (*p* > 0.001). Education levels 0–2 (*F*(3, 27) = 13.168) are the most salient indicators in the clustering for “0 portions,” while education levels 5–8 (*F*(3, 27) = 19.380) are the most salient in the clustering for “1 to 4 portions,” emphasizing their importance as determinants of daily FV consumption.

**TABLE 9:** Results of the ANOVA test.

	CMS	df	EMS	df	Statistic	Value	Sig.
0 portions							
Levels 0–2	5.940	3	0.451	27	F	13.168	0.000
Levels 3–4	5.869	3	0.459	27	F	12.787	0.000
Levels 5–8	5.373	3	0.514	27	F	10.450	0.000
1–4 portions							
Levels 0–2	6.296	3	0.412	27	F	15.297	0.000
Levels 3–4	6.109	3	0.432	27	F	14.129	0.000
Levels 5–8	6.829	3	0.352	27	F	19.380	0.000
5+ portions							
Levels 0–2	6.730	3	0.363	2.466	Welch	7.174^a^	0.094
Levels 3–4	6.778	3	0.358	2.418	Welch	4.936^a^	0.142
Levels 5–8	5.739	3	0.473	2.296	Welch	2.919^a^	0.242

Abbreviations: CMS, clustering mean square; EMS, error mean square.

^a^ Asymptotically F distributed.

The Bonferroni test was used in the post hoc analysis to determine significant differences between the clusters for the consumption categories “0 daily portions” and “1–4 daily portions,” stratified by educational level (Table 10). The test results differ between the countries living in clusters C_1 and C_3. In cluster C_1, the proportion of people who reported no daily consumption of FV was highest in all education levels (0–2, 3–4, and 5–8). In clusters C_2 and C_3, the proportion of people who stated that they did not consume FV daily was also considerable at all education levels.

**TABLE 10:** Results of post hoc test.

Dependent variable		*I*	*J*	*I*–*J*	SE	Sig.	95% CI	
							LL	UL
0 portions	Levels 0–2	1	2	0.9891	0.495	0.335	−0.4204	2.3988
			3	−2.7096*	0.495	0.000	−4.1193	−1.3001
			4	−0.8086	0.364	0.209	−1.8445	0.2272
		2	3	−3.6988*	0.672	0.000	−5.6110	−1.7868
			4	−1.7978*	0.582	0.028	−3.4538	−0.1419
		3	4	1.9010*	0.582	0.018	0.2451	3.5570
	Levels 3–4	1	2	1.1110	0.499	0.208	−0.3109	2.5329
			3	−2.5149*	0.499	0.000	−3.9368	−1.0931
			4	−0.9973	0.367	0.068	−2.0422	0.0475
		2	3	−3.6259*	0.677	0.000	−5.5547	−1.6972
			4	−2.1083*	0.587	0.008	−3.7787	−0.4381
		3	4	1.5175	0.587	0.092	−0.1527	3.1879
	Levels 5–8	1	2	1.0865	0.529	0.298	−0.4188	2.5909
			3	−2.5510*	0.529	0.000	−4.0560	−1.0462
			4	−0.6604	0.388	0.604	−1.7663	0.4454
		2	3	−3.6371*	0.717	0.000	−5.6785	−1.5958
			4	−1.7464	0.621	0.054	−3.5143	0.0214
		3	4	1.8906*	0.621	0.031	0.1228	3.6585
1–4 portions	Levels 0–2	1	2	0.0645	0.473	0.099	−1.2819	1.4110
			3	2.5422*	0.473	0.000	1.1958	3.8887
			4	1.6065*	0.348	0.001	0.6171	2.5959
		2	3	2.4776*	0.642	0.004	0.6513	4.3041
			4	1.5419	0.556	0.059	−0.0398	3.1237
		3	4	−0.9357	0.556	0.622	−2.5175	0.6460
	Levels 3–4	1	2	0.4931	0.485	0.099	−0.8869	1.8732
			3	2.1689*	0.485	0.001	0.7889	3.5490
			4	1.8468*	0.356	0.000	0.8328	2.8610
		2	3	1.6757	0.658	0.101	−0.1962	3.5477
			4	1.3536	0.569	0.149	−0.2675	2.9749
		3	4	−0.3220	0.569	0.099	−1.9432	1.2991
	Levels 5–8	1	2	0.8658	0.438	0.349	−0.3800	2.1117
			3	2.0232*	0.438	0.001	0.7774	3.2690
			4	2.0727*	0.322	0.000	1.1573	2.9882
		2	3	1.1573	0.594	0.370	−0.5326	2.8473
			4	1.2068	0.514	0.159	−0.2566	2.6704
		3	4	0.04952	0.514	0.099	−1.4140	1.5130

Abbreviations: *I*, cluster number of cases; *I*–*J*, mean difference; *J*, cluster number of cases; LL, lower limit; SE, standard error; UL, upper limit.

* The mean difference is significant at the 0.05 level.

Cluster C_2 had lower consumption rates than C_1 across all educational levels. In clusters C_3 and C_4, the prevalence of non‐consumption of FV was even higher, especially in C_4, where non‐consumption was most common (all education levels: 0–2, 3–4, 5–8). The statistical significance of these results, confirmed at the p‐level, calls for a comprehensive dialog on nutrition interventions and public health strategies. The countries with the lowest FV consumption across all education levels are in clusters C_3 (Latvia and Romania) and C_4 (Denmark, Lithuania, Luxembourg, and the Netherlands), which is a clear conclusion. Looking at daily consumption, which includes one to four portions of FV, compared to education levels 0–2, 3–4, and 5–8, the differences between countries in C_1 and those in C_3 and C_4 are clearly visible. The countries in C_1 show a higher prevalence of this dietary behavior, while the countries in C_3 and C_4 show an opposite pattern.

A clear difference was observed between clusters C_2 and C_3, with C_2 showing higher participation in this practice across education levels 0–2. Twenty‐three countries were categorized in C_1 and two in C_2 (France and Ireland), characterized by a higher consumption of 1–4 daily portions of FVs compared to the other clusters at all education levels.

## 4 Discussion

The study of K‐Means clusters for daily consumption of FVs, stratified by educational level in an impressive number of 31 European countries, has revealed the existence of four extraordinarily different clusters. The first cluster (C_1) includes Austria, Belgium, Bulgaria, Croatia, Cyprus, the Czech Republic, Estonia, Finland, Germany, Greece, Hungary, Iceland, Italy, Malta, Norway, Poland, Portugal, Serbia, Slovakia, Slovenia, Spain, Sweden, and Turkey—with each nation making its own vibrant contribution to this diverse ensemble. The second cluster (C_2) is comparatively smaller, but includes the prominent and respected nations of France and Ireland. The only members of the third cluster (C_3) are Latvia and Romania, while the fourth cluster (C_4) comprises the dynamic quartet of Denmark, Lithuania, Luxembourg, and the Netherlands. It is noteworthy that the countries in clusters C_1 and C_4 show considerable similarities and form a link amidst their different characteristics, while the countries in clusters C_2 and C_3 show the strongest contrasts. Latvia and Romania, which belong to C_3 and are located in the eastern and southern regions, are a focus of the analysis.

A pattern is emerging across Europe: Eastern European countries have the largest deficit in FV, a trend that can be observed at all levels of education. In stark contrast, countries in Western, Southern, and Central Europe exude a vibrant zeal and unprecedented enthusiasm for these vital, health‐promoting foods. A comprehensive systematic review of global vegetable consumption and supply in 2020 clearly shows the skewed picture of vegetable consumption in Europe: a remarkable 46% is accounted for by Western, 39% by Central and 37% by Southern areas (Kalmpourtzidou et al. 2020). Empirical evidence shows a large discrepancy and a significant deficit in average FV consumption in Eastern European countries compared to the laudable WHO benchmarks, which lag strikingly behind their European counterparts (Lock et al. 2005). It is clear that despite extensive efforts by advocates, there is a large gap between ideal consumption and actual dietary habits in relation to FVs in Eastern European countries.

The study included a representative selection of European countries (Austria, Belgium, Finland, France, Germany, Iceland, Ireland, Italy, Luxembourg, Malta, Norway, the Netherlands, Poland, and Sweden), which provided socio‐economic and educational diversity for the cluster analysis. Furthermore, the intertwining of the upscale regional Nordic diet in countries such as Iceland, Finland, Norway and Denmark with the prestigious mediterranean diet in countries such as Cyprus, Greece, Italy, Malta, Portugal, Spain, and Turkey is undeniably a cornerstone for the emergence of these clusters. The Nordic diet advocates enriching the diet with healthy culinary gems such as hearty fruits, nutrient‐rich vegetables, fresh omega‐rich fish, hearty oats, nutritious nuts, healthy legumes, as well as barley, rye, and health‐conscious low‐fat dairy products (Mithril et al. 2013). There is no doubt that this issue is of paramount importance. Celebrated as a timelessly sustainable and predominantly plant‐centered gastronomic ethos, the Mediterranean diet remains an iconic way of eating in modern times (Burlingame and Dernini 2011; Dernini and Berry 2015).

In‐depth literature reviews have found a correlation between the decline in educational attainment and vegetable consumption, particularly in low to middle income countries (Baars et al. 2019; Hall et al. 2009). Conversely, it is evident that an increase in educational attainment is often accompanied by an increase in vegetable consumption (Eurostat and Forti 2017). In a study conducted across EU countries in 2017, 27% of participants reported consuming fruit twice a day, while 37% reported eating fruit once a day. Equally remarkable is that 23% of respondents consume vegetables twice a day, while a staggering 40% include these nutrient‐rich marvels in their diet every day. Countries with Mediterranean climates, such as Spain, Italy, and Portugal, have the highest fruit consumption, which is a testament to their vibrant and rich culinary traditions, closely followed by dedicated eaters in Ireland and Luxembourg. In terms of vegetable consumption, Ireland, Belgium, Italy, Portugal, and Luxembourg are the undisputed champions of healthy eating. In stark contrast, many Eastern European countries, with the notable exception of Romania, are worryingly far behind. They have the lowest vegetable consumption rates on the continent, a daunting challenge vividly illustrated in the comprehensive and insightful data from Eurostat and Forti (2017).

The country clusters identified in this study are consistent with the patterns observed in Eurostat (2017) data, indicating consistent consumption patterns over time. In 2020, the European Commission introduced financial support measures for the agri‐food sector to promote FV consumption across the EU (European Commission 2020; Morosi 2016). These measures aim to improve dietary habits, with a particular focus on the school and work environment.

## 5 Limitations and Future Recommendations

This study has several limitations that should be considered when interpreting the results. First, the use of self‐reported survey data may introduce biases related to recall accuracy and social desirability when reporting dietary habits. Second, the cross‐sectional nature of the data limits our ability to establish causal relationships between educational attainment and dietary behavior. Third, while cluster analysis provides valuable insights at the national level, it may overlook important subnational differences that are influenced by factors such as income differences, urban–rural disparities, or age‐specific consumption trends. Furthermore, we were unable to account for potential confounding factors such as local food policies, cultural dietary habits, or economic accessibility of FVs that could further explain consumption patterns. Future research could address these limitations by incorporating longitudinal studies to better understand the temporal relationships between education and diet. More detailed analysis at a regional or demographic level could help to mitigate environmental misconceptions and identify targeted intervention points. Combining quantitative approaches with qualitative methods, such as nutritional behavior surveys or policy analysis, would place the results of clustering in a broader context. Finally, exploring how education‐based nutrition programs could be tailored to specific country clusters, particularly those with the lowest FV consumption, could provide more effective public health strategies. These advances would strengthen the evidence base for tackling dietary inequalities across Europe.

## 6 Conclusions

The analysis of daily FV intake in 31 countries in the European region, examined as a function of education level, shows that countries tend to group themselves not only according to the amount of FV consumption but also according to more general patterns of overall intake. Those with an education level of 0–2 were found to have a higher prevalence of “0 portions” in their daily diet. In contrast, those with an education level of 5–8 were found to be more likely to consume 1–4 portions daily, reflecting an association between higher education levels and more frequent consumption of FVs. A detailed examination of country profiles within the most similar clusters shows that nations from Western, Northern and Southern Europe are clustered together in groups characterized primarily by higher socioeconomic and educational levels. In addition, the traditional dietary patterns prevalent in these countries, such as the Mediterranean or Nordic diet, appear to play a role in linking countries with similar profiles.

## Author Contributions

**Mustafa Enes Işıkgöz:** conceptualization (equal), data curation (equal), formal analysis (equal), investigation (equal), methodology (equal). **Mahir Arslan:** writing – original draft (equal), writing – review and editing (equal).

## Ethics Statement

Publicly available statistical data from the Eurostat database was used for this study. The data is accessible to the general public and does not contain any personal or sensitive information. As the study was conducted with anonymized, aggregated data, ethics committee approval was not required. The analysis was conducted in accordance with European Union data protection regulations and adhered to established standards for transparency and integrity in research.

## Conflicts of Interest

The authors declare no conflicts of interest.

## Data Availability

The datasets generated and/or analyzed during the current study are available in the [EUROSTAT] repository, [https://doi.org/10.2908/hlth_ehis_fv3e].
